# Compliance with the Very Integrated Program (VIP) for Smoking Cessation, Nutrition, Physical Activity and Comorbidity Education Among Patients in Treatment for Alcohol and Drug Addiction

**DOI:** 10.3390/ijerph16132285

**Published:** 2019-06-28

**Authors:** Karen Hovhannisyan, Michelle Günther, Rie Raffing, Maria Wikström, Johanna Adami, Hanne Tønnesen

**Affiliations:** 1 Clinical Health Promotion Centre, WHO Collaborating Centre for Implementation of Evidence-based Clinical Health Promotion, Faculty of Medicine, Lund University and Addiction Centre Malmö, Region Skåne, Södra Förstadsgatan 35, 4th floor, SE 205 02 Malmö, Sweden; 2 Skånevård Kryh, Medicon Village, Region Skåne, 223 81 Lund, Sweden; 3 Clinical Health Promotion Centre, WHO Collaborating Centre for Evidence-based Health promotion in Hospitals and Health Services, The Parker Institute, Bispebjerg & Frederiksberg Hospital, University of Copenhagen, Nordre Fasanvej 57-59, Entr. 5, 2000 Frederiksberg, Denmark; 4 Student Health, Malmö University, Neptuniplan 7, 21118 Malmö, Sweden; 5 President Office, Sophiahemmet University, Box 5605, 114 86 Stockholm, Sweden

**Keywords:** compliance, lifestyle intervention, meeting adherence, self-efficacy, treatment programs, patient education

## Abstract

Meeting adherence is an important element of compliance in treatment programmes. It is influenced by several factors one being self-efficacy. We aimed to investigate the association between self-efficacy and meeting adherence and other factors of importance for adherence among patients with alcohol and drug addiction who were undergoing an intensive lifestyle intervention. The intervention consisted of a 6-week Very Integrated Programme. High meeting adherence was defined as >75% participation. The association between self-efficacy and meeting adherence were analysed. The qualitative analyses identified themes important for the patients and were performed as text condensation. High self-efficacy was associated with high meeting adherence (ρ = 0.24, *p* = 0.03). In the multivariate analyses two variables were significant: avoid complications (OR: 0.51, 95% CI: 0.29–0.90) and self-efficacy (OR: 1.28, 95% CI: 1.00–1.63). Reflections on lifestyle change resulted in the themes of Health and Wellbeing, Personal Economy, Acceptance of Change, and Emotions Related to Lifestyle Change. A higher level of self-efficacy was positively associated with meeting adherence. Patients score high on avoiding complications but then adherence to the intervention drops. There was no difference in the reflections on lifestyle change between the group with high adherence and the group with low adherence.

## 1. Introduction

Patients with alcohol and drug addiction often have additional risky lifestyle behaviours, e.g., smoking, poor nutrition and physical inactivity, as well as comorbidity; all these have a social gradient and add to the already high substance-induced morbidity and pre-mortality [1,2,3]. Effective health promotion aiming at those factors is therefore relevant to offer to this disadvantaged patient group as an integrated part of addiction treatment. However, lifestyle change is a complex process influenced by many factors related to the programme, the therapist and the individual person.

To meet the multiple needs related to lifestyle change among patients with alcohol and drug addiction, the individually tailored Very Integrated Program (VIP) has been translated from the 6-week intensive Danish Gold Standard Programme (GSP) on smoking cessation intervention with an approximately 30% continuous quit rate [4,5,6] for alcohol intervention, nutritional intervention, physical activity or combinations of these [7].

The intervention employs an Operational Model to facilitate successful lifestyle change, which incorporates the theories of Motivational Interviewing [8], Decisional Balance and the Trans-Theoretical Model of Change [9], all of which are related to the process of behavioural change [10]. A Cochrane review suggest that using only one theoretical approach may be insufficient for patients who are attempting lifestyle changes in relation to clinical treatment and argues that an intervention should include both measures to increase motivation, intensive behavioural support as well as nicotine replacement therapy in the case of smoking cessation to get the best results [11].

The prediction of a successful lifestyle intervention can have a major impact even for patients with substance use and severe mental diseases [12]. Among other issues the meeting adherence has been reported to be a strong predictor for successful outcomes [4,13,14,15]. Nevertheless, the factors associated with meeting adherence have been less investigated. The level of motivation and self-efficacy tend to be relevant and have been reported to be an important factor for prompting engagement into the process of change [8,9,16]. Although the self-efficacy itself is found not to be a robust predictor among patients with alcohol addiction [17].

According to Albert Bandura self-efficacy is defined as a person’s confidence in his or her intrinsic ability to accomplish goals [18] and is often measured by a visual analogue scale or a Likert scale similar to the one widely implemented for pain measurement. Central to the theory of self-efficacy are the person’s own expectations of efficacy, which determine the initiation of a coping behaviour, how much effort he or she will put into it, and for how long the effort will be sustained when confronted with barriers and obstructions. Reflections on the advantages and disadvantages of changing [19] may also be of importance for meeting adherence, and individuals with high meeting adherence might hypothetically have different reflections than those with low meeting adherence.

The primary aim of this study was to evaluate the possible association between the patients’ self-assessment of importance of avoiding complications, importance of changing lifestyle and their level of self-efficacy and adherence to the VIP intervention among patients undergoing treatment for alcohol and drug addiction. The secondary aims were to identify other possible factors of importance for meeting adherence.

## 2. Materials and Methods

**Figure 1:**
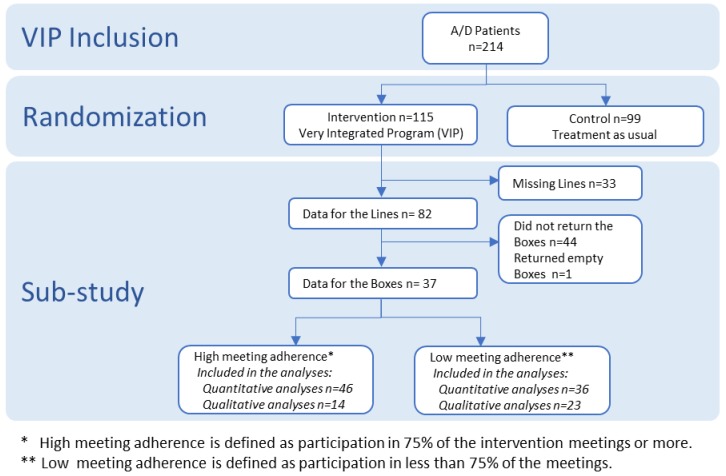
Study Profile. VIP: Very Integrated Program.

This study is a sub-study of a randomised controlled trial which will be reported at a later stage. This study includes the patients in the intervention group of the randomised study. The RCT was a clinical trial called VIP that took place in two addiction centres in Malmö, Sweden (Figure 1). The aim of the VIP-Study was to assess the effect of adding health promotion activities to treatment of alcohol and drug addiction. Results on the effect of the intervention are planned to be reported at a later stage [20]. The eligible participants were persons aged 18 or over and diagnosed with alcohol or drug addiction according to the ICD-10 criteria by the specialists at the Addiction Centre Malmö (4 units) and the Integrated Community Care Centre (one unit), Psychiatry Skåne, Sweden. The inclusion criteria for VIP was alcohol and drug addiction in accordance to ICD-10 criteria among patients who also had at least one risky lifestyle behaviour (daily smoking, daily physical activity less than 30 min, overweight and risk of malnutrition) and at least one comorbidity (diabetes, cardiovascular, respiratory and liver diseases). In VIP 115 patients were allocated to the intervention group. This present sub-study includes a total of 82 patients with alcohol or drug addiction who had completed The Line Tool for the quantitative study and 37 who completed The Box Tool were included in the qualitative study (Figure 1).

**Figure 2:**
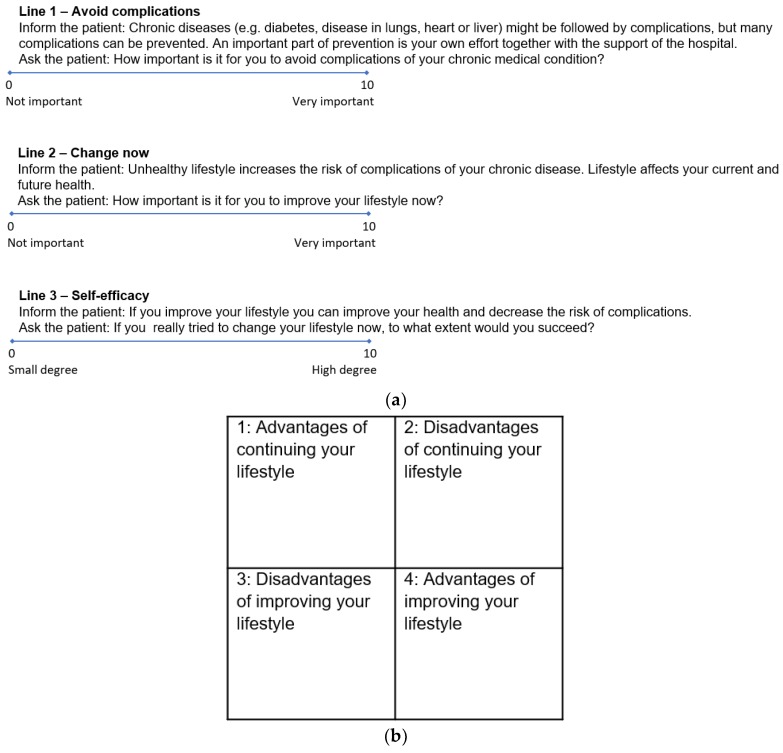
(**a**) The Line Tool (0–10 cm) [10], (**b**) The Box Tool (part 1–4) [10].

After obtaining informed consent, the VIP intervention group filled in The Line Tool as visual analogue scales [21] (Figure 2a). The lines are known from the motivational interviewing technique, where they have been validated [8]. The scale exists both in a version from 1–10 cm and a version from 0–10 cm. In this study we used the version from 0–10 which is similar to the visual analogue scales which is used in the clinical setting worldwide. The patients also structured their reflections as 1–4 in The Box Tool (Figure 2b).

The VIP intervention was based on the Operational Model and translated from the GSP intervention. GSP includes motivational dialogues, an educational programme with five sessions aimed at risky lifestyle behaviours and an interactive workshop on comorbidity directed at patients and relatives. This programme involves six face-to-face meetings with a trained counsellor, each one being approximately an hour in duration. The Line Tool used in this model made it possible to rank the patients’ self-efficacy in achieving the change amongst others, while The Box Tool, besides assessing the advantages and disadvantages of the change, allowed patients to reflect upon the presence of ambivalence towards the change [10]. The patients kept the forms throughout the intervention as a reminder of their reflections. In this way we integrated qualitative research methods in the larger context of the clinical trial. It is argued in the developing debate on process evaluation that this is fruitful because empirical data add to the understanding of how an intervention is experienced by the participants, and how participants interact with the intervention under the influence of other factors such as circumstances, attitudes, beliefs, social norms and resources [22,23].

### 2.1. Data Collection

#### 2.1.1. Quantitative Data

We collected the following information at inclusion of patients in the VIP study: Age, duration of addiction, gender, socioeconomic and lifestyle factors, comorbidity, self-evaluated quality of life via the Short Form Health Survey (SF-36) [24] (Table 1) and meeting adherence defined as attendance to the meetings. In total the intervention included five meetings over six weeks approximately of one-hour duration each. Meeting attendance was recorded by the clinician performing the intervention. Adherence was dichotomised into high (≥75%) and low attendance (<75%) of sessions as high adherence has been shown to triple quit rates [25]. The data for lines came from measures on the VAS scale 0–10 cm (Figure 2a). SF-36 is used to measure the self-reported quality of life [24]. The questions in SF-36 are focused around two main domains, the physical and mental health [24]. For example, the first item reads “In general, would you say your health is…” with the scoring options “Excellent, Very good, Good, Fair, Poor”, and item nine a reads “These questions are about how you feel and how things have been with you during the past month. How much time during the past month: Did you feel full of life?” having the answering options “All of the time, Most of the time, A good bit of the time, Some of the time, A little of the time, None of the time” [24].

The reliability and validity of the survey has been tested among many patient populations including patients with alcohol and drug addiction [26,27]. They reported Cronbach’s Alpha ≥ 0.7. In a Swedish context it has also proven a high reliability with a Cronbach’s Alpha ≥ 0.8. [28].

#### 2.1.2. Qualitative Data

The qualitative data consisted of the patients’ reflections on advantages and disadvantages regarding lifestyle intervention as described above in the Box Tool (Figure 2b).

### 2.2. Data Analysis

#### 2.2.1. Quantitative Analyses

First, we used the Spearman’s correlation test (ρ) to examine the association between The Line Tool components measuring the patients’ perceived importance of the following categories: avoid complications (Line 1), immediate change (Line 2) and self-efficacy (Line 3) and meeting adherence.

To analyse differences between the groups with high- and low attendance, we conducted univariate analyses and used the Chi-square test for the dichotomous variables and Mann–Whitney U test for continuous variables. The significance level was set at 0.05, two-sided. All the predictive variables such us Complications, Change, Self-efficacy, Age, Years of addiction and Sex were entered together into the multivariable logistic regression analysis for identifying factors of significance using odds ratio (OR) with 95% confidence interval (CI).

For quantitative analyses we used the IBM SPSS Statistics software, version 22 [29].

#### 2.2.2. Qualitative Analyses—Systematic Text Condensation

The handwritten answers of the patients in The Box Tool were converted to the digital form of an Excel sheet and imported into NVivo qualitative data analysis software, version 11 [30] for coding. Some of the answers had no direct relation to the questions and were therefore excluded from the analyses.

The data was analysed using Kirsti Malterud’s approach of systematic text condensation [31]. Systematic text condensation consists of 4 steps: Total impression of all answers and identifying preliminary themes, Coding by identifying and sorting meaning units, Condensation into code groups and Synthesising the condensates into a story grounded in the empirical data.

KH and RR independently read the patient reflections thoroughly to get a total impression and to identify preliminary themes. These themes were largely identical such as “Health” and “Economy”, but there were also some discrepancies. For example, the preliminary theme of “Social life/family” was only identified by one of the coders and some themes such as “Weight” and “More alert” were included in the overall theme of “Health”. Subsequently, the data was systematically reviewed for meaning units, which gave rise to codes that were then separated into thematic groups and a condensate was developed. This was done by RR and then the thematic groups and sub-groups were discussed by KH and RR until consensus was reached. The condensates were then synthesised, and descriptions and concepts were developed by RR to clarify the study question. Since the research objective was to identify important factors for adherence, the data were sorted in high and low adherence and analysed accordingly. The two analyses were then checked for divergent themes.

### 2.3. Comprehensiveness of the Available Data

#### 2.3.1. Quantitative Data: Sample Size Estimation

The number was calculated using: *n* = (Z_2α_ + Z_β_)^2^ × (P_1_(1 − P_1_) + P_2_(1 − P_2_))/(P_1_ − P_2_)^2^, where 2α = 5% => Z_2α_ = 1.96 and 1 − β = 80% => β = 20% => Z_β_ = 0.84. P_1_ = 75% and P_2_ = 45% => *n* = 38 in each group of high and low attendance [32]. P_2_ = 45% (the lowest estimate for attendance). P_1_ = 75% (the highest estimate for attendance).

#### 2.3.2. Qualitative Data: Data Saturation

In qualitative research, the indication of when to stop collecting data is the methodological principle of data saturation; as data is being collected, patterns and themes will emerge and begin to reproduce themselves. When this happens, it is estimated that no further data is needed because the data is “saturated” [33]. In traditional qualitative interview studies, data saturation is expected to be reached after approximately 12 interviews [34]. In our case data was collected from all 37 patients and as expected several major themes were identified in the data.

### 2.4. Ethical Issues

The VIP study has been approved by the Ethical Review Board (Dnr2010/470) in accordance with the Swedish Data Protection Agency and the protocol was registered in clinicaltrials.gov (NCT01414907). The patients were included after informed consent.

## 3. Results

### 3.1. Quantitative Data

We found that only self-efficacy (Line 3) was positively associated with meeting adherence, ρ = 0.24, *p* = 0.03 which was significant. The patients’ perceptions of importance for changing lifestyle immediately (Line 2) and avoiding complications (Line 1) was not found to be statistically significant.

In the univariate analyses the only significant difference between the two groups with high and low adherence were the perception of self-efficacy, which remained significant in the multivariate analyses (OR: 1.23; 95% CI: 1.01–1.51). The importance of avoiding complications, although not significant in the univariate analyses, became significant in the multivariable model (OR: 0.51; 95% CI: 0.29–0.90) (Table 2).

### 3.2. Qualitative Data

We identified 4 major themes and 11 sub-themes (Table 3). There were no substantial differences between the answers of the group with high adherence and the group with low adherence. The same themes and sub-themes were present, and only the subtheme of “Continuing current lifestyle restrains individuals in poor economy” was referred to a little less frequently in the group with high adherence.

Considering the major themes and sub-themes identified through the analysis, there were evident patterns in patient reflections. These patterns drew a common picture of patients, of whom many were very aware of how their lifestyle influenced their health and wellbeing.

They saw improving lifestyle as an advantage because it would improve their health and they were aware that if they continued their current lifestyle it would not only cause poor health and future health risks but possibly also a premature death.

It was also evident for the patients that lifestyle change would have an impact on their personal economy. They expected to be restrained in poor economy if they continued their current lifestyle and to improve their personal economy if they improved their lifestyle.

The patients expressed a strong acceptance of change finding no advantages of continuing their current lifestyle and finding no disadvantages of improving their current lifestyle.

Finally, it became clear that the patients related strong emotions to lifestyle change. They found that maintaining status quo was easier than changing routine. They also found positive social implications of lifestyle improvement and especially emphasised improved contact with their children. They associated their current lifestyle with positive emotions and expected lifestyle improvement to cause negative emotions.

## 4. Discussion

We found that meeting adherence during the 6-week VIP intervention was positively associated with a high pre-intervention score for self-efficacy. This is similar to results of another study that found that positive expectations had improved effect on treatment retention [35] but on the other side, an older review found that changes in the self-efficacy and the benefit from self-efficacy on outcome was heterogeneous [36]. We also identified several themes in the patients’ reflections on the advantages and disadvantages of lifestyle change: Health and Well-being, Personal Economy, Acceptance of Change, and Emotions Related to Lifestyle Change. These themes were identical in the groups with high and low adherence.

It is a surprising result, that patients score high on avoiding complications (Line 1), but then their adherence to the intervention drop, although this result was not significant.

The positive correlation between the patients’ expectations of their own efficacy and the duration they adhered to the intervention is in line with Bandura’s theory [18], but despite the wide discussion of self-efficacy in the literature, we found no other studies about self-efficacy and integrated health promotion intervention on patients with alcohol and drug addiction. However, our results are to some degree supported by other studies. A previous review about the effect of self-efficacy on various health care interventions concluded that self-efficacy is a powerful predictor for better intervention outcomes, especially regarding smoking secession and, to some extent, physical activity and weight control [37]. A more recent study found that high confidence (equivalent to self-efficacy, Line 3) was associated with a positive outcome regarding both drinking and smoking [38] while another study associated the low self-efficacy with a relapse among drug users [39]. On the other side, an older study found that the lower score of self-efficacy at the baseline was associated with higher rates of abstinence after 6 months among the patients with alcohol addiction, but the same group of patients had two-fold increase in self-efficacy score during this period [40] this was also supported by another study [41]. Nevertheless, a study by Romero et al. reported the intermediate self-efficacy score to be associated with the better outcomes among the patients with the drug addiction, whereas the low and high scores predicted a inferiors results [42].

Other studies have reported contradictory results on participant adherence to a 12-week physical activity programme. However, many other factors, such as health literacy and numeracy, may also impact self-reporting [43], but no clear cut-offs, mediators and moderators for the effect of health literacy have been determined for clinical use [44].

### 4.1. Methodological Implications

Qualitative methods can help us understand the clinical context of complex interventions and identify unexpected mechanisms [45]. In this study the qualitative answers shed more light on the results obtained from the quantitative measures. For example, the patients’ responses that they find avoiding complications very important is unfolded in their descriptions of which complications they are afraid to experience and what consequences it will have for their life. However, on this background it is unexpected to observe, that they less frequently come to the meetings.

The way patients interact with the intervention is influenced by their circumstances, attitudes, beliefs, social norms and resources [22,23]. It is interesting that both the group with high and the group with low meeting adherence are aware that continuing their current lifestyle might cause poor health and future health risks including possible premature death and improving their lifestyle will improve their health. A high awareness of these issues does not seem to influence the motivation to attend the meetings of the intervention since assessing it as important to avoid complications of comorbidity on the line scale is associated with lower meeting adherence.

In many ways these results capture the complexity of patient reflections as they stand on the brink of lifestyle change. Advantages and disadvantages coexist. For example, there seems to be an acceptance of change, because when asked, the patients see no advantages of continuing their unhealthy lifestyle and no disadvantages of improving their lifestyle. The patients point to health and wellbeing as major advantages of changing lifestyle and they expect their personal economy to improve. At the same they express strong emotions of barriers to lifestyle change. The routines of their current lifestyle have a strong hold of them, they associate positive emotions to their current lifestyle and expect that changing lifestyle will cause negative emotions. These factors might contribute to an explanation of why an intervention might (not) work, for whom, and under what circumstances [45].

These major themes from the reflections of Health and Wellbeing, Personal Economy, Acceptance of Change, and Emotions Related to Lifestyle Change may also be of importance for the general support and facilitation of the changing process among this group of patients with alcohol and drug addiction. The clear messages on personal economy and the high acceptance of change seem easy to include and revisit to drive support for the patients’ process of change.

### 4.2. Clinical Implications

A special focus should be put on how positive emotions such as inner calm and anxiety relief are experienced as an advantage of current lifestyle with addiction and that negative emotions are expected to be a disadvantage of changing this lifestyle. Additionally, the expression of emotions related to changing of lifestyle should be an important element for the staff supporting the change; they should acknowledge both that staying with the status quo is easier than changing routine and that the current lifestyle is associated with the positive emotions while improving lifestyle is expected to cause negative emotions. While acknowledging the expectations of the patients, intervention staff should also rely on evidence in the area.

### 4.3. Research Implications

In addition to co-morbidity the VIP-programme intervenes on the four risk factors of smoking, physical inactivity, overweight and malnutrition. Evidence on the association between risk reduction and positive emotional and mental state already exist in several areas.

Interestingly, a systematic review with meta-analyses from 2014 shows that smoking cessation is associated with reduced depression, anxiety and stress after the period of withdrawal symptoms [46]. Therefore, issues of smoking cessation for patients with a psychiatric diagnosis should not be avoided [47], but on the contrary, encouraged [12].

A review from 2019 shows how exercise has positive effects on brain health including decreased risks of depression and stress in of patients with neurological and mental illnesses [48]. In the general population, physical activity also has a positive effect on anxiety [48] and the evidence is still growing for a general positive effect of physical activity on mental health [49].

We have not been able to identify reviews on the influence of improved nutrition on well-being in this patient group, and this knowledge gap needs to be investigated in future research.

In the VIP-study, the recruited patients were already in treatment for alcohol and drug abuse, and therefore these risk factors were not included in the VIP-programme. However, a trial by Berman et al. shows that participants with a problematic alcohol intake who reduce this intake reported better wellbeing including lower stress, better social life satisfaction and lower rated of depressed mood at 12 months follow up. There was no difference in the group of drug users [50].

### 4.4. Suggestions for Future Research

The results of this study can generate hypotheses for future studies and suggest explanations that can inform future interventions [22] where such hypotheses can be tested. It would be clinically relevant to investigate if seeking to obtain a possible outcome triggers lifestyle change more than seeking to avoid a negative outcome. For example, if seeking to obtain improved health triggers lifestyle change more than seeking to avoid a premature death.

The time perspective is another clinically relevant area for investigation. Studies have shown that patients are motivated to quit drinking in the perioperative period to avoid postoperative complications such as cardiopulmonary complications, bleeding episodes and infections [51]. Our results indicate, that the patients in this study are not motivated to change lifestyle by reducing their risk of complications to their chronic medical condition. To optimise lifestyle intervention, it would be relevant to investigate if decision making of lifestyle change is associated with the expected time perspective (short term or long term) of the benefit of the change.

To summarise we could assume that the surprising quantitative findings reflects the patients’ coexistence of positive and negative expectations to lifestyle change. The patients are concerned about complications, but at the same time they do not have the resources handle the expected negative emotions that come with the change and therefore they do not come to the meetings. On the other hand, the ones who rate themselves high on self-efficacy manage to set aside the expected negative emotions and focus on the expected positive outcomes of improved health and economy and therefore come to the meetings to continue their lifestyle change.

### 4.5. Lessons Learned

(1) There is a discrepancy between what patients say and know about the importance of avoiding complications and what they do to avoid them when it comes to adhere to an intervention programme.

(2) Patients have negative expectations towards the emotional side of lifestyle change, though there is evidence for the contrary.

### 4.6. Strengths and Limitations

Among the strengths of this study was the general homogeneity of the included patients who furthermore also received the same VIP intervention irrespective of their self-efficacy score.

Even though 33 (29%) of the patients from the original VIP study were not included in this sub-study due to missing data, the power estimate illustrated that the 82 included patients were sufficient for the quantitative analyses. In the same manner, the analyses for data saturation confirmed that the data from the 37 completed boxes would be sufficient for including into the qualitative analyses.

The box was easy to complete and did not require extensive involvement from the staff. On the other hand, it prompted shorter answers which could be challenging for in depth data extraction. When filling in the box, the patient was supposed to begin with the question “Advantages of the current lifestyle” and finish with answering the question “Advantages of changing”. It was supposed to structure the considerations and ambivalence related to the process towards improving lifestyle change [10]. This could also predispose the patients to answer in a certain way. However, since the project staff did not interfere with completing the boxes this possible bias was considered low.

The two statistically significant variables from the quantitative analyses were avoiding complications and self-efficacy. The risk of a type I error could be reduced by repeating the study. On the other hand, the non-significant results of other variables could be attributed to the type II error and may have reached a significant level if the study population was even larger.

The study only included patients from addiction treatment organisations in Malmö, Skåne region, which might have different demographics compared not only with other countries but also with other regions within Sweden. This implies that a similar study conducted in a different location might garner different findings.

In this study meeting adherence was defined in relation to the 6-week intervention. Longer- or shorter-interventions could thus show different results.

## 5. Conclusions

We found that when patients score high on wanting to avoid complications, their meeting adherence drops, and a high level of self-efficacy was positively associated with high adherence to the intervention meetings among patients with alcohol and drug addiction. The negative association between avoiding complications and meeting adherence is surprising and should be further investigated in future studies. Recognising the self-efficacy as a predictive factor for better intervention compliance in this group should be accepted with precaution. There was no difference in the identified themes of pros and cons of the patients with high and low adherence. In general, the patients showed solid concerns about their health and wellbeing as well as their personal economy. They were ready for change, but also displayed strong emotions regarding the process of change.

## Figures and Tables

**Table 1:** Participant characteristics.

Variables	Total (*n* = 82)
Line 1—Avoid Complications (median (range))	10 (2–10)
Line 2—Change now (median (range))	9 (3–10)
Line 3—Self-efficacy (median (range))	7 (0–10)
Age in years (median (range))	53 (27–72)
Years of addiction (median (range))	25 (1–60)
Men (*n* (%))	49 (60%)
Alcohol addiction (*n* (%))	44 (54%)
Living alone (*n* (%))	64 (78%)
Higher educational level (*n* (%))	29 (35%)
No housing (*n* (%))	13 (16%)
Unemployed * (*n* (%))	19 (23%)
Number of health determinants (≥2)	53 (65%)
Number of co-morbidities (≥2)	24 (29%)
Physical health (median (range))	54 (0–100)
Mental Health (median (range))	38 (0–100)

* By unemployed we do not refer to students, homemakers or retired.

**Table 2:** Comparison of the characteristics of the groups with high and low meeting attendance.

Variables	Total *n* = 82	High MA * *n* = 46	Low MA * *n* = 36	OR (95% CI) Unadjusted	OR (95% CI) Adjusted
Line 1—Avoid Complications (median (range))	10 (2–10)	10 (2–10)	10 (5–10)	0.75 (0.54–1.07)	0.51 (0.29–0.90)
Line 2—Change now (median (range))	9 (3–10)	9 (6–10)	9 (3–10)	1.24 (0.90–1.70)	1.69 (0.91–3.13)
Line 3—Self-efficacy (median (range))	7 (0–10)	8 (1–10)	6 (0–10)	1.23 (1.01–1.51)	1.28 (1.00–1.63)
Age in years (median (range))	53 (27–72)	54 (27–72)	52 (27–72)	1.01 (0.97–1.04)	1.02 (0.97–1.07)
Years of addiction (median (range))	25 (1–60)	27 (3–57)	21 (1–60)	1.01 (0.98–1.04)	1.02 (0.98–1.06)
Men (*n* (%))	49 (60%)	27 (55%)	22 (45%)	0.90 (0.37–2.20)	0.95 (0.33–2.67)

* MA is meeting adherence.

**Table 3:** Reflections on advantages and disadvantages of lifestyle change.

Themes (Capital Letters), Sub-Themes (Bold), Condensations, Authentic Illustrative Quotations (Italic)
HEALTH AND WELLBEING
**Improved health is an advantage of improving lifestyle**
The patients expressed, that they expect a major advantage of improving their lifestyle to be that they will feel better, that their health will be better, and that their strength will improve—both physically and mentally. As examples they mention getting rid of high blood pressure, asthma will improve, and weight loss. They also expect lifestyle change will help them recover, feel refreshed, get a better self-image and be more positive. They also expect to get a better old age and live longer. “*Feel better physically and mentally and improved strength*”.
**Continuing current lifestyle causes poor health and future health risks**
The major disadvantage experienced by the patients of their current lifestyle is their deteriorated physical and mental health. They are often ill, they have a cough and high blood pressure. They feel in bad shape; they are overweight and fear not being able to breathe. Their stress and concern increase, and they struggle with anxiety. They are aware of the risk of getting COPD, a stroke, cancer, chronic diseases or problems with their heart. They know, that their current lifestyle does not lead to a long and healthy life. “*I do not give myself the chance to live a longer and healthier life*”.
**Continuing current lifestyle might lead to a premature death**
The patients are aware that a disadvantage of their current lifestyle is, that it might shorten their lifespan and that their children consequently would be without a parent should they die prematurely. “*Running the risk of a premature death*”.
PERSONAL ECONOMY
**Improving lifestyle will improve personal economy**
Spending money on other things is seen as an advantage of changing lifestyle by the patients. They expect that it will improve their living conditions, and that they perhaps would have a surplus. “*More money over to other things*”.
**Continuing current lifestyle restrains individuals in poor economy**
Living with poor economy is experienced as a disadvantage of the patients’ current lifestyle. It costs them too much and they live a poor and depleted life. “*It costs too much*”.
ACCEPTANCE OF CHANGE
**There are no advantages of continuing current lifestyle**
The patients find it difficult to see advantages of their current lifestyle. “*There are no advantages, actually*”.
**There are no disadvantages of improving lifestyle**
The patients see no disadvantages of changing their lifestyle. “*There are no disadvantages*”.
EMOTIONS RELATED TO LIFESTYLE CHANGE
**Status quo is easier than changing routine**
The patients must not change lifestyle and they see this as an advantage. They find it difficult to begin something new and to make an effort to break a habit. They find it convenient not to do anything, not to care or to think about their flaws. Their current lifestyle feels safe to them because some of them fear changes. They know, that changing lifestyle will demand a commitment from them, they think nicotine abstinences will cause anxiety and they will lose the opportunity to sleep all day. “*Escape changes and all the effort it implies, peace of mind*”.
**Positive social implications of lifestyle improvement especially improved contact with children**
The patients believe, that they will become more social if they change lifestyle. They think of how they could do more together with their children and how they would have a longer future together. They also envision how they might find a partner, how they would not smell of smoke all the time, how they might become a role model for others and support family members in smoking cessation. “*My daughter worries so much*”.
**Current lifestyle is associated with positive emotions**
The current lifestyle of the patients makes them calm inside and helps them suppress their anxiety. They experience that they become less aggressive, and it makes them feel good. They also think of it as a pleasant social activity. “*Makes me calm inside and suppresses my anxiety*”.
**Improving lifestyle is expected to cause negative emotions**
The patients expect lifestyle change to influence their mood negatively. They think they will get a lot of “do’s”, for example about what they eat and with whom they socialise. In that way, they imagine they would feel their life would be limited compared to their current life. “*Bad mood*”.
